# Ruminal microbial metagenomes and host transcriptomes shed light on individual variability in the growth rate of lambs before weaning: the regulated mechanism and potential long-term effect on the host

**DOI:** 10.1128/msystems.00873-24

**Published:** 2024-08-20

**Authors:** Fan Hu, Yan Cheng, Bing Fan, Wei Li, Bingsen Ye, Zhiwu Wu, Zhiliang Tan, Zhixiong He

**Affiliations:** 1 CAS Key Laboratory for Agro-Ecological Processes in Subtropical Region, National Engineering Laboratory for Pollution Control and Waste Utilization in Livestock and Poultry Production, Hunan Provincial Key Laboratory of Animal Nutritional Physiology and Metabolic Process, Institute of Subtropical Agriculture, The Chinese Academy of Sciences, Changsha, Hunan, China; 2 University of Chinese Academy of Sciences, Beijing, China; 3 Hulun Buir State Farm Technology Development, Hailar, China; 4 Hulun Buir State Farm Tenihe Farm, Hulun Buir, China; The University of Maine, Orono, Maine, USA

**Keywords:** metagenome, transcriptome, pre-weaning lamb

## Abstract

Weaning weight is a reflection of management during the breastfeeding phase and will influence animal performance in subsequent phases, considered important indicators within production systems. The aims of this study were as follows: (i) to investigate variability in the growth rate among individual lambs from ewes rearing single or twin lambs fed with two different diets and (ii) to explore the molecular mechanisms regulating the growth rate and the potential long-term effects on the host. No significant change in lamb average daily gain (ADG) was observed in litter size and diet treatment, and there were large variations among individual lambs (ranging from 0.13 to 0.41 kg/day). Further analysis was conducted on serum amino acids, rumen fermentation characteristics, rumen metagenomics and transcriptome, and hepatic transcriptome of lambs with extremely high (HA; *n* = 6) and low (LA; *n* = 6) ADG. We observed significant increases in serum lysine, leucine, alanine, and phenylalanine in the HA group. The metagenome revealed that the HA group presented a higher rumen propionate molar proportion via increasing gene abundance in the succinate pathway for propionate synthesis. For the rumen transcriptome, higher expressed gene sets in the HA group were mainly related to rumen epithelial growth, including cytokine–cytokine receptor interaction, Jak-STAT signaling pathway, and adherens junction. For the liver transcriptome, the upregulated KEGG pathways in the HA group were primarily associated with fatty acid degradation, glyoxylate and dicarboxylate metabolism, cholesterol metabolism, and the immune system. This research suggests that preweaning lambs with high ADG may benefit from rumen development and enhanced liver metabolic and immune function.

**IMPORTANCE:**

There is accumulating evidence indicating that the early-life rumen microbiome plays vital roles in rumen development and microbial fermentation, which subsequently affects the growth of young ruminants. The liver is also vital to regulate the metabolism and distribution of nutrients. Our results demonstrate that lambs with high average daily gain (ADG) enhanced microbial volatile fatty acid (VFA) metabolism toward rumen propionate and serum amino acid (AA) production to support host growth. The study highlights that high ADG in the preweaning period is beneficial for the rumen development and liver energy metabolism, leading to better growth later in life. Overall, this study explores the molecular mechanisms regulating the growth rate and the potential long-term effects of increased growth rate on the host metabolism, providing fundamental knowledge about nutrient manipulation in pre-weaning.

## INTRODUCTION

Due to the rapid population growth and increase in revenue across the globe, the trends of dietary needs shifted toward high-quality animal-based protein products (meat, eggs, and dairy), and these demands will probably continue for the next three decades (1, 2). To meet the growing demand and increase profit, selecting the most efficient animal or animals with maximum growth potential is of incredible interest to the livestock industry. It is widely accepted that the transition phase is a critical window period to manipulate visceral organ development and metabolic processes and invoke epigenetic programming (3). Average daily gain (ADG) in early life has a lifetime impact on productivity, and an increased growth rate is associated with later health status and growth performance, thus improving the industry profit (4, 5). The optimal growth rate of heifers reduces the age at first calving and increases the productivity and longevity of these animals (6). In addition, there is increasing evidence that an increase in the preweaning growth of young calves was positively correlated with milk production in the first lactation (5). Unfortunately, the knowledge gap regarding the underlying mechanisms of growth rate in early life is even larger for lambs, with still scarce information available. It is also unclear how preweaning ADG levels could improve long-term performance by regulating host function.

For ruminants, the rumen and liver are major digestive and metabolic organs that play a prominent role in nutrient metabolism and energy supply. As the volatile fatty acid (VFA) producer, the rumen accounts for 70% of the total energy requirements. Ruminal microbiota has been reported to be key drivers of energy conversion for the maintenance and growth of the host (7), and the orientation of VFA production will largely determine the amount of energy in the rumen epithelium and liver (8). The close and constant interaction between the host and rumen microbiota is a vital prerequisite for underpinning host health and optimal productivity. Efficient nutrient delivery, absorption, and metabolism depend upon the transition of rumen function during weaning from the preruminant to ruminant stages (9). The liver adapts to nutrient patterns absorbed due to rumen development and is responsible for modulating and distributing these nutrients through crosstalk with multiple tissues (10). Previous studies have found that lipid metabolism and immune response in the liver are key processes associated with nutrient metabolism in pigs, beef cattle, sheep, and poultry in the fattening period (11).

With the advancement of sequencing techniques, we can now better understand the microbiome function through metagenome analysis and the biological functions of upregulated or downregulated genes through transcriptomic analysis. This improved knowledge of molecular and functional mechanisms enables us to gather biological information about the underlying factors influencing the ADG of preweaning lambs. In our study, we specifically selected animals with extreme ADG to gain a deeper understanding of growth rates in early life. We focused on preweaning lambs to investigate the responsive mechanisms and potential long-term effects of rumen microbiota and biological functions using 16S rRNA gene analysis, rumen metagenomics, transcriptome analysis, and hepatic transcriptome analysis.

## MATERIALS AND METHODS

### Animal, diets, and experimental design

Twenty Hulunbuir lactating ewes (red headed sheep) rearing single or twin lambs were raised with two different concentrates and allocated into four groups: T-1 = lactating ewes rearing twin lambs fed with concentrate diet 1 and rapeseed straw, *n* = 5; S-1 = lactating ewes rearing a single lamb fed with concentrate diet 1 and rapeseed straw, *n* = 4; T-2 = lactating ewes rearing twin lambs fed with concentrate diet 2 and rapeseed straw, *n* = 7; S-2 = lactating ewes rearing a single lamb fed with concentrate diet 2 and rapeseed straw, *n* = 4 (Fig. S1). The concentrate diet 1 contained 57.86% corn, 13.46% barley, 9.2% wheat bran, 3.56% soybean meal, 8.13% cottonseed meal, 5.25% premix, and 2.54% beet molasses. The concentrate diet 2 consisted of 30% corn, 30% barley, 20% rapeseed meal, and 20% wheat. The main nutrient components of the diets are listed in Table S1. The experiment began when the lambs were 1 month old and lasted for 6 weeks. The dams with single lambs were fed with 0.55 kg concentrate and 2 kg rapeseed straw. Ewes rearing two or more lambs have higher nutritional requirements than ewes rearing a single lamb, so twin-rearing ewes were fed with 0.7 kg concentrate and 2 kg rapeseed straw. All the experimental lambs were breastfed, and these cohorts had uniform dietary regimes, rearing conditions, and similar genetic backgrounds. Lambs were weighed before morning feeding at the beginning and the last day of the experimental period, and ADG was calculated: ADG = (final weight – initial weight)/42 days.

### Sample collection and determination

Blood samples were collected through the jugular vein using 10-mL vacutainers with no anticoagulant (SANLI Medical Technology Development Co., Ltd., Changsha, China) before the morning feeding. After centrifugation at 3,000  ×  *g* at 4°C for 15  minutes, the supernatants were collected, aliquoted into 1.5-mL microcentrifuge tubes, and stored at −20°C to determine biochemistry parameters and free amino acid profiles. The levels of glucose (GLU), triglyceride (TG), cholesterol (CHOL), low-density lipoprotein (LDL), high-density lipoprotein (HDL), blood urea nitrogen (BUN), and total protein (TP) in collected serum samples were measured using an automatic biochemical analyzer (Cobas c311, Roche, Basel, Switzerland). The serum samples were centrifuged at 3,000  ×  g for 5  minutes, and the supernatants were collected and mixed with 1  mL of 8% sulfosalicylic acid solution. After centrifugation at 13,000  rpm for 10  minutes, the supernatant was filtered through 0.22-µm filters and then analyzed by an ion-exchange amino acid analyzer (L8800, Hitachi, Tokyo, Japan).

The entire rumen content was removed, homogenized, and a subsample was transferred into a 15-mL tube, snap-frozen in liquid nitrogen, and stored at −80°C for bacterial analysis by 16S rRNA gene and metagenomics. Ruminal content was collected and stored at −20°C to determine the level of volatile fatty acids by gas chromatography (Agilent 7890A, Agilent Inc., Palo Alto, CA). Ruminal tissues from the ventral sac region were washed in normal saline and stored at −80°C for RNA extraction and subsequent transcriptomic analysis.

The liver was rapidly sampled from each left lobe, immediately frozen in liquid nitrogen, and then stored at −80°C until it was used for RNA extraction.

### DNA extraction, 16S rRNA gene sequencing, and data analysis

Microbial DNA was extracted from rumen content samples using the E.Z.N.A. stool DNA Kit (Omega Bio-tek, Norcross, GA, USA). The V3–V4 regions of bacterial 16S rRNA genes were amplified using primers 341F (5′-CCTAYGGGRBGCASCAG-3′) and 806R (5′-GGACTACNNGGGTATCTAAT-3′). Amplicons that separated from 2% agarose gels were purified by the AxyPrep DNA Gel Extraction Kit (Axygen Biosciences, Union City, CA). The PCR products were used to construct the Illumina Pair-End library on the Illumina MiSeq platform (Shanghai Biozeron Co., Ltd).

For data analysis, operational taxonomic units (OTUs) were clustered with a 97% similarity cutoff using UPARSE. The most abundant sequences within each OTU, which aligned with the SILVA database, were designated as representative sequences by the RDP classifier. The α-diversity was measured to determine the richness and diversity of the bacterial community, including the Chao, ACE, and Shannon diversity indices. The Bray–Curtis distance metrics and Adonis analysis were carried out to assess the bacterial community similarity among the 12 samples. The Wilcoxon test was carried out to examine the significant differences in the phylum and genus levels.

### Shotgun metagenomic sequencing and analysis

Shotgun metagenomic sequencing libraries were constructed and sequenced at Shanghai Biozeron Biological Technology. Trimmomatic and Burrows–Wheeler aligner (BWA) tools were used to filter out adapter contaminants, low-quality reads, and *Ovis aries* adulterants from the raw sequence reads. Subsequently, clean sequence reads were assembled using MegaHit with “--min-contig-len 500” parameters. The open reading frames (ORFs) of assembled contigs were predicted using Prodigal (v2.6.3), and ORFs were generated as a set of unique genes after clustering using CD-HIT. The unique-gene set was searched against the KEGG databases using BLASTX to identify the proteins and retrieve their functional annotations. Based on the KEGG Orthologs (KO) results, the specific functions and pathways of each sample were obtained using the pathway mapped by the annotated genes using the KEGG database. The KO genes with an average TPM >2 were used for downstream analysis.

### Transcriptome sequencing and data analysis

The TRIzol method was used to extract total RNA from 12 ruminal epithelium samples and 12 liver tissue samples. Subsequently, the concentration and integrity of the RNA were determined by using a NanoDrop spectrophotometer (Thermo Fisher Scientific, MA, USA) and Agilent 2100 Bioanalyzer (Agilent Technologies, CA, USA). The raw data were filtered with SOAPnuke (v1.5.2) by (i) removing adapter contamination; (ii) removing reads whose unknown base (“N” base) ratio is more than 5%; and (iii) removing reads whose low-quality base ratio is more than 20%; afterward, clean reads were obtained and stored in FASTQ format. The clean filtered reads were aligned to the reference genome (GCF_002742125.1_Oar_rambouillet_v1.0) using HISAT. Transcripts per kilobase of exon model per Million mapped reads (TPM) were used to estimate the gene expression in each sample. Within-group differential gene analysis was performed using DESeq2. The differentially expressed genes (DEGs) were selected by the threshold values: FDR < 0.05 and absolute fold change  ≥ 2. The KEGG database was used to conduct pathway enrichment annotation of DEGs. Since the number of differentially expressed genes observed was small, our focus should be limited not only to significant DEGs but also to the genes that play a major role. The gene set enrichment analysis (GSEA) method could avoid choosing arbitrary cutoffs and accumulate subtle expression changes in the same group of gene sets for studying functional enrichment between two biological groups. The GSEA was used to identify the KEGG pathways with the most significant changes in expression in the LA and HA groups (|NES|  >  1, *P* < 0.05, FDR *q*-value < 0.25).

### Statistical analysis

The rumen fermentation characteristics, serum biochemical parameters, and free amino acid profile data analysis were performed on IBM SPSS version 22.0 (SPSS, Chicago, IL, USA). The *t*-test was used to determine the differences in ADG, serum biochemical parameters, free amino acid profile, and rumen VFAs between the two groups. The Wilcoxon test with FDR correction was applied to determine the relative abundance of microbial phyla, genera, OTUs, and KEGG pathways. Statistical significance was defined at *P* < 0.05, and the tendency was considered at 0.05 ≤ *P* < .10. Analyses of the matrix between rumen VFA, rumen microbial VFA pathway genes, and liver transcriptome were performed by the Mantel test using the linkET package in R.

## RESULT

### ADG distribution

**Fig 1:**
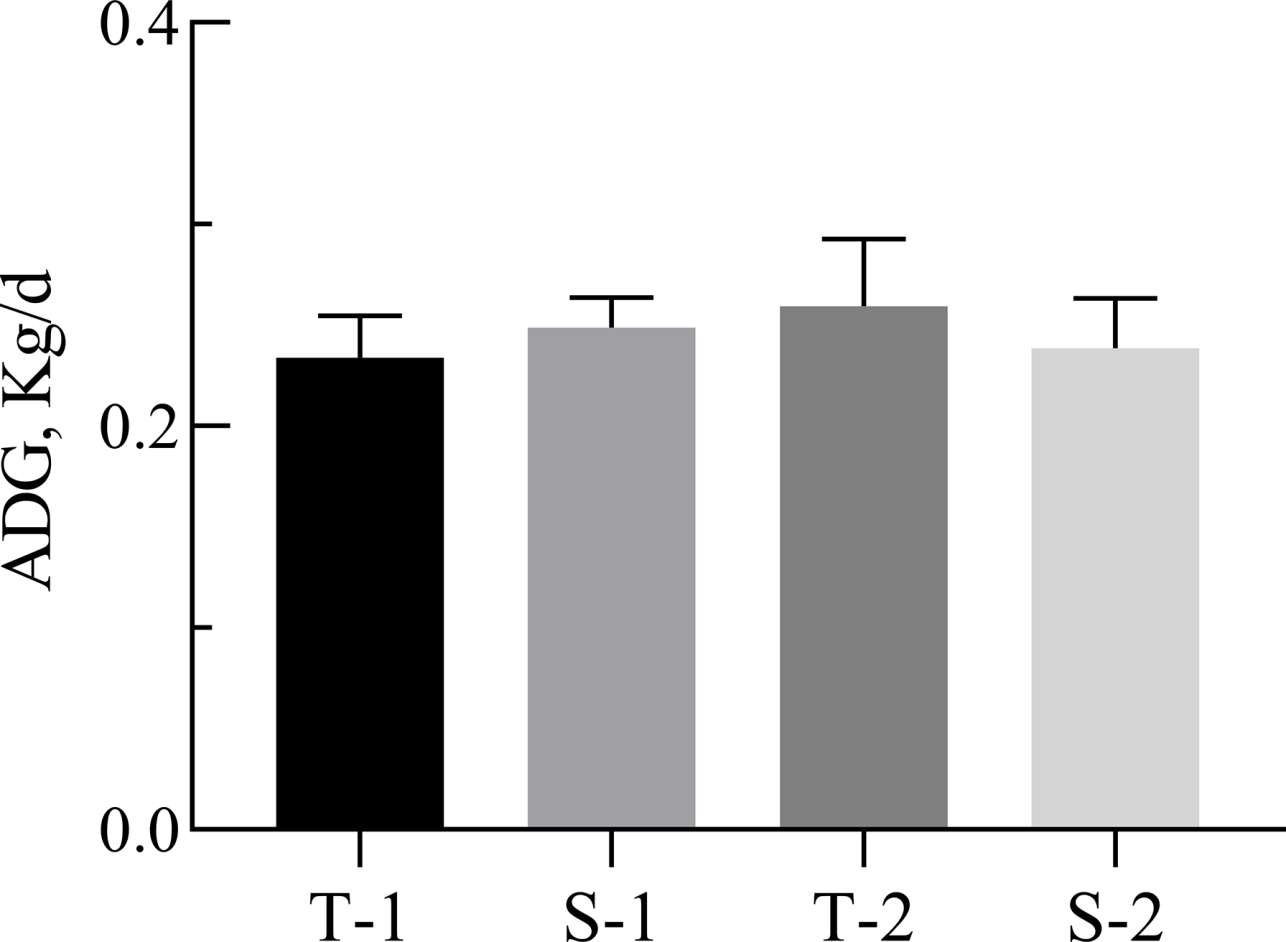
The effect of maternal nutrition levels (MN) and being born as a singleton or twins (ST) on average daily gain (ADG) of preweaning lambs. T-1 = lactating ewes rearing twin lambs fed with concentrate diet 1 and rapeseed straw, *n* = 5; S-1 = lactating ewes rearing a single lamb fed with concentrate diet 1 and rapeseed straw, *n* = 4; T-2 = lactating ewes rearing twin lambs fed with concentrate diet 2 and rapeseed straw, *n* = 7; S-2 = lactating ewes rearing a single lamb fed with concentrate diet 2 and rapeseed straw, *n* = 4. Values are presented as mean ± SEM. *P* values of two-way ANOVA (MN = 0.793, ST = 0.922, and MN × ST = 0.549).

**Fig 2:**
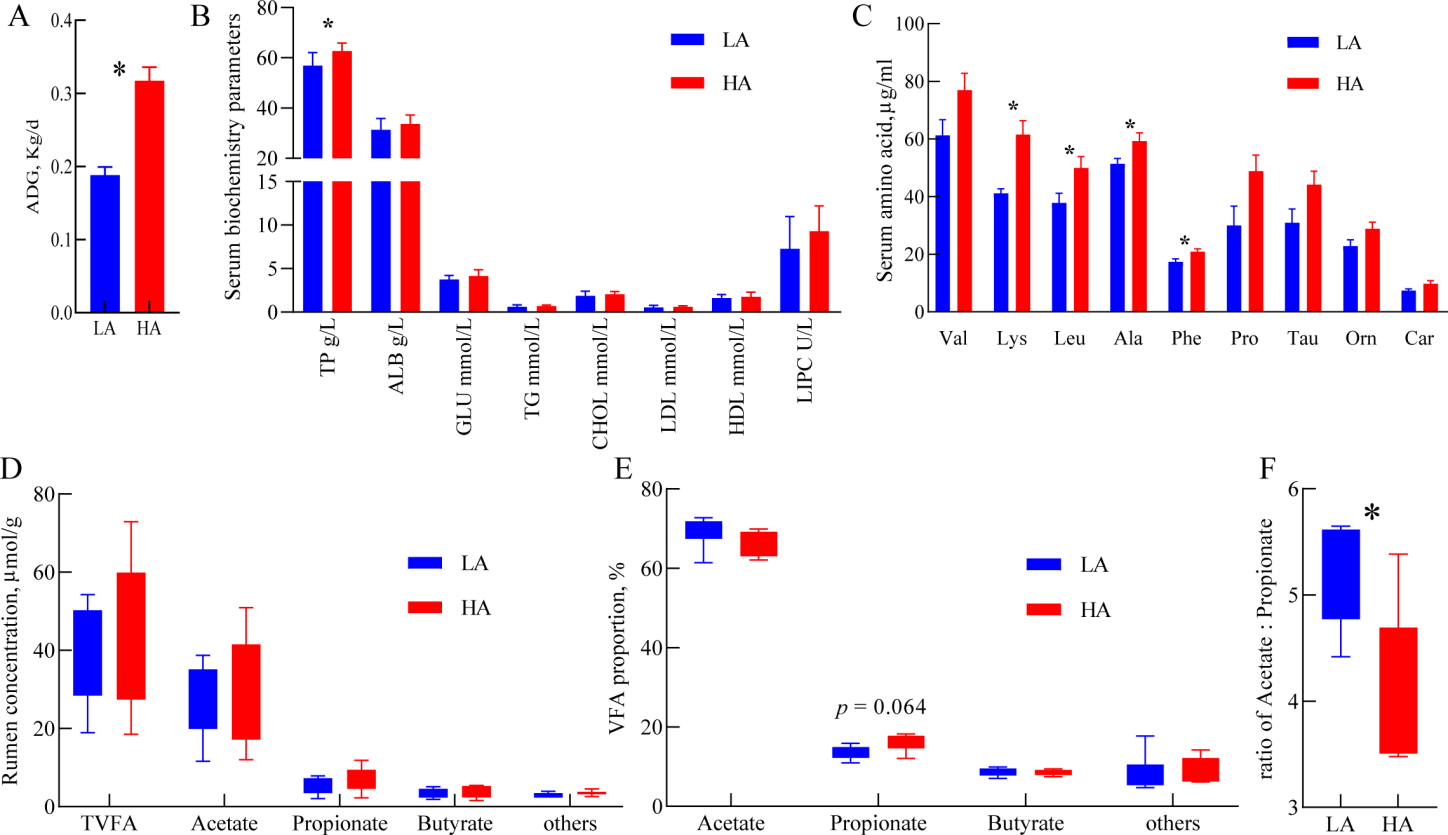
Comparisons of the ADG phenotype, biochemical parameters, and free amino acid profile in the serum and rumen fermentation parameters between the low (LA) and high (HA) ADG groups. (**A**) ADG phenotype in lambs; (**B**) serum biochemical parameters; (**C**) serum free amino acid profile; Comparisons of the concentrations (**D**) and proportion (**E**) of rumen VFA; (**F**) the ratio of acetate to propionate. The differences between the two groups were tested by *t*-test (*n* = 6 per group). The bars represent mean  ±  SEM. *, *P*  <  0.05. ALB, albumin. GLU, glucose. TG, triglyceride. CHOL, cholesterol. LDL, low-density lipoprotein. HDL, high-density lipoprotein. LIPC, hepatic lipase. Lys, lysine. Leu, leucine. Ala, alanine. Phe, phenylalanine. Tau, taurine. Val, valine. Orn, ornithine. Car, carnosine. Pro, proline.

There were no significant changes in lamb ADG among the four treatments (Fig. 1). Substantial variations among individual lambs were observed based on ADG (ranging from 0.13 kg/day to 0.41 kg/day), and details of lambs are provided in Table 1. Twelve of the most extreme samples from lambs with high and low ADG were selected for later microbial and transcriptomic analyses. Based on the individual ADG, lambs with relatively higher (ADG = 0.32 kg/day) and lower ADG (ADG = 0.19 kg/day) were assigned to the HA and LA group, respectively (Fig. 2A).

**TABLE 1:** Details of lambs with low or high average daily gain^*d*^

Diet of lactating ewes	Lambs	Group	Initial weight	Final weight	Weight gain	ADG^*c*^
Concentrate 2 + rapeseed straw	Twin	LA^*a*^	13.1	18.7	5.6	0.13
Concentrate 2 + rapeseed straw	Single	LA	13.9	21.8	7.9	0.19
Concentrate 1 + rapeseed straw	Twin	LA	9.7	17.9	8.2	0.20
Concentrate 2 + rapeseed straw	Twin	LA	10.2	18.7	8.5	0.20
Concentrate 1 + rapeseed straw	Twin	LA	13.3	21.9	8.6	0.20
Concentrate 1 + rapeseed straw	Twin	LA	10.1	18.7	8.6	0.20
Concentrate 2 + rapeseed straw	Single	—
Concentrate 1 + rapeseed straw	Single	—
Concentrate 1 + rapeseed straw	Single	—
Concentrate 2 + rapeseed straw	Twin	—
Concentrate 2 + rapeseed straw	Twin	—
Concentrate 1 + rapeseed straw	Single	—
Concentrate 2 + rapeseed straw	Single	—
Concentrate 1 + rapeseed straw	Twin	—
Concentrate 2 + rapeseed straw	Twin	HA^*b*^	17.1	29.3	12.2	0.29
Concentrate 1 + rapeseed straw	Single	HA	13.1	25.4	12.3	0.29
Concentrate 2 + rapeseed straw	Single	HA	11.1	23.6	12.5	0.30
Concentrate 1 + rapeseed straw	Twin	HA	12.1	24.8	12.7	0.30
Concentrate 2 + rapeseed straw	Twin	HA	12.2	29.4	17.2	0.41
Concentrate 2 + rapeseed straw	Twin	HA	11.1	24.1	13	0.31

^*a*^ LA = low average daily gain group (*n* = 6).

^*b*^ HA = high average daily gain group (*n* = 6).

^*c*^ ADG = average daily gain, kg/day.

^*d*^ Twelve of the most extreme samples from lambs with high and low ADG were selected for later microbial and transcriptomic analysis in the study. Other samples, marked "—," were rejected for further analysis.

### Comparison of serum biochemical parameters and free amino acid profile in lambs with divergent ADG

The lambs in the HA group had a higher total protein (TP) concentration (*P* = 0.041) than those in the LA group (Fig. 2B). No significant differences (*P* > 0.05) were observed between the two groups in serum ALB, GLU, TG, CHOL, LDL, HDL, and LIPC levels. For serum-free amino acid profiles (Fig. 2C), the HA group showed a markedly enhanced lysine (Lys; *P* = 0.007), leucine (Leu; *P* = 0.042), alanine (Ala; *P* = 0.044), and phenylalanine (Phe; *P* = 0.044) content compared with the LA group. Likewise, the taurine (Tau; *P* = 0.073), valine (Val; *P* = 0.076), ornithine (Orn; *P* = 0.090), carnosine (Car; *P* = 0.087), and proline (Pro; *P* = 0.056) concentrations tended to be increased in the serum of lambs from the HA group compared with the LA group. The other features of serum amino acids (Table S2) showed similar concentrations between the two groups (*P* > 0.05).

### Lambs with divergent ADG shows distinct ruminal fermentation characteristics

The concentration of total VFA, acetate, propionate, and butyrate (*P* > 0.05) remained unchanged (Fig. 2D). Compared with the LA group (Fig. 2E and F), the HA group had a lower ratio of acetate to propionate (*P* = 0.023) and tended to be higher in the proportion of propionate (*P* = 0.064). There were no significant discrepancies (*P* > 0.05) between the two groups in the molar proportion of acetate, butyrate, and other VFAs.

### Taxonomic distribution of rumen bacteria in lambs with different ADG via 16S rRNA gene sequencing

**Fig 3:**
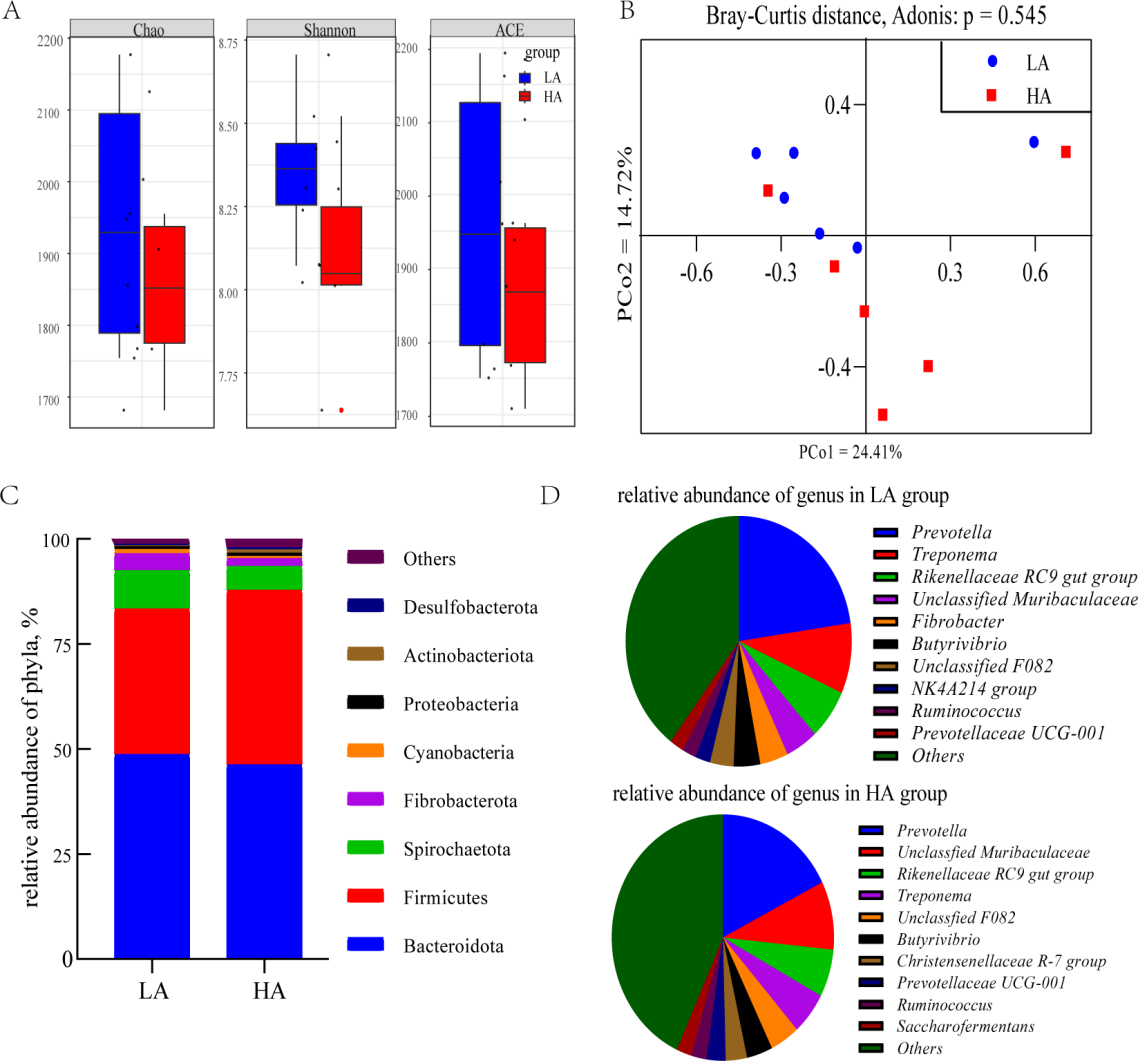
Taxonomic profile of rumen bacteria in the low (LA) and high (HA) ADG groups. (**A**) Alpha diversity analysis of rumen microbiota (Chao, Shannon, and ACE). (**B**) Principal coordinate analysis (PCoA) based on Bray–Curtis distances. Adonis analysis showed no significant differences between the two groups (*P*  =  0.545); relative abundances of the bacterial community at the phylum (**C**) and genus levels (**D**) between the two groups based on the 16S rRNA sequencing data (*n* = 6).

There was a total of 644,602 high-quality reads, with an average of 50,966  ±  1,975 reads per sample via 16S rRNA sequencing. The Chao, Shannon, and ACE indexes in the HA group were similar to those in the LA group (*P* > 0.05, Fig. 3A). The Adonis based on Bray–Curtis distance showed no significant difference in rumen bacteria between the two groups (Adonis, *P* = 0.545, Fig. 3B). The phylogenetic composition of the rumen microbial community was dominated by Bacteroidetes, Firmicutes, Spirochaetota, and Fibrobacterota at the phylum level (Fig. 3C), representing more than 95% of taxa detected in rumen content samples. At the genus level, we only listed the top 10 bacterial genera whose relative abundances were greater in each group (Fig. 3D). Our results showed that *Prevotella* (LA vs HA: 22.68% vs 17.61%) was the dominant genus in both groups. However, there were no significant differences between the two groups in the relative abundances of the other phyla and genera.

### Functional profile of the rumen metagenome of lambs with different ADG

To mechanistically probe the functional capacity of the rumen microbiome, we performed shotgun metagenomic sequencing in 12 samples from the rumen content. Metagenomic sequencing of 12 samples generated a total of 1,914,046,902 raw reads. After quality control and removing host genes, a total of 1,870,343,004 clean reads were retained.

**Fig 4:**
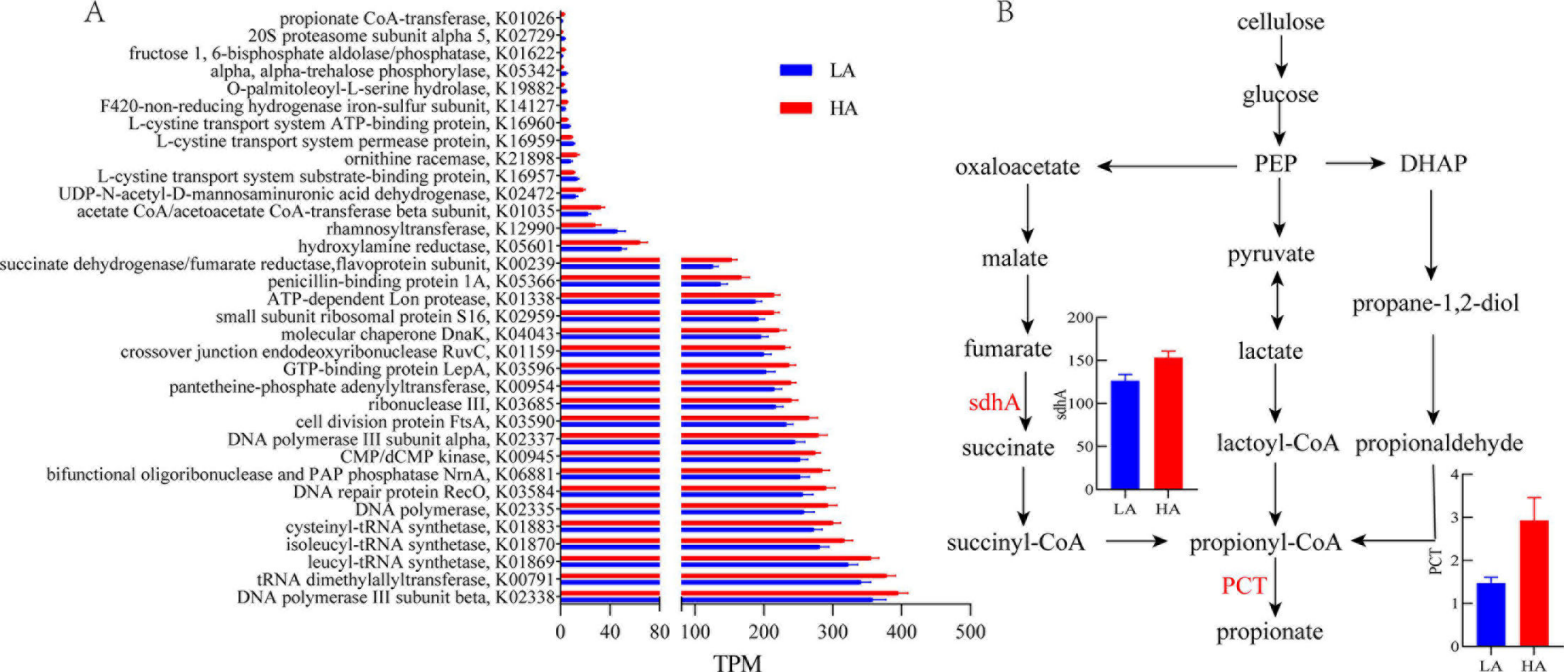
Comparisons of microbial functions in metagenomic analysis between the low (LA) and high (HA) ADG groups. (**A**) Significantly different KO enzymes; (**B**) metabolic pathways involved in the propionate production pathway. Red font and arrows indicated upregulated enzyme genes in the HA group. The Wilcoxon test with Benjamini–Hochberg (HB) adjustment was carried out to compare the significant differences in the relative abundance of KO enzymes (TPM) between the two groups (*n* = 6).

Out of the nonredundant gene set, 3,536,483 (36.66%) genes could be classified into KEGG orthology, and a total of 3,146 unique KO were identified. For KEGG pathway level 3, the expression of the bacterial secretion system (*P* = 0.015), furfural degradation (*P* = 0.011), and zeatin biosynthesis (*P* = 0.045) were significantly increased in the HA group (Fig S2). For KEGG profiles, 34 differentially expressed KO genes are listed in Table S3. Among different KO genes, K02338 (DNA polymerase III subunit beta), K00791 (tRNA dimethylallyltransferase), K01869 (leucyl-tRNA synthetase), K01870 (isoleucyl-tRNA synthetase), and K01870 (isoleucyl-tRNA synthetase) were the most abundant (Fig. 4A). Given the crucial role of ruminal propionate in the phenotype changes of lambs, genes encoding enzymes that are directly involved in propionate metabolism were analyzed for their abundance in LA and HA groups. Interestingly, the HA group increased the abundance of the succinate dehydrogenase flavoprotein subunit in the succinate pathway (K00239, sdhA, EC:1.3.5.1, *P* = 0.030) compared with the LA group. Furthermore, lambs in the HA group also had a higher abundance of the propionate CoA-transferase (K01026, PCT, EC:2.8.3.1, *P* = 0.043) than those in the LA group, but the other genes were unchanged (Fig. 4B).

### Transcriptomes of the rumen and liver of lambs with different ADG

To investigate the differences in the host gene transcriptional changes between the two groups, we performed transcriptome sequencing on total RNA from 24 samples (12 lambs with two tissues). A total of 61.84 million clean reads were generated from rumen and liver samples. The overall mapping rates ranged from 94.31% to 96.73% (Table S4). About 14,384 and 14,589 expressed genes were detected in the rumen and liver samples, respectively.

DEGs were identified using the criteria of at least a twofold difference in expression and FDR < 0.05. Since no DEG was observed between groups in the rumen samples, GSEA was used to further identify the KEGG pathways or functional components of genes with the most significant changes. As for the KEGG-based list (Table S5), higher expression gene sets in the HA group were mainly associated with rumen epithelial growth, such as cytokine–cytokine receptor interaction (NES = 1.692, *P* < 0.001, FDR = 0.075), Jak-STAT signaling pathway (NES = 1.675, *P* < 0.001, FDR = 0.079), and adherens junction (NES = 1.5852, *P* < 0.001, FDR = 0.11939).

**Fig 5:**
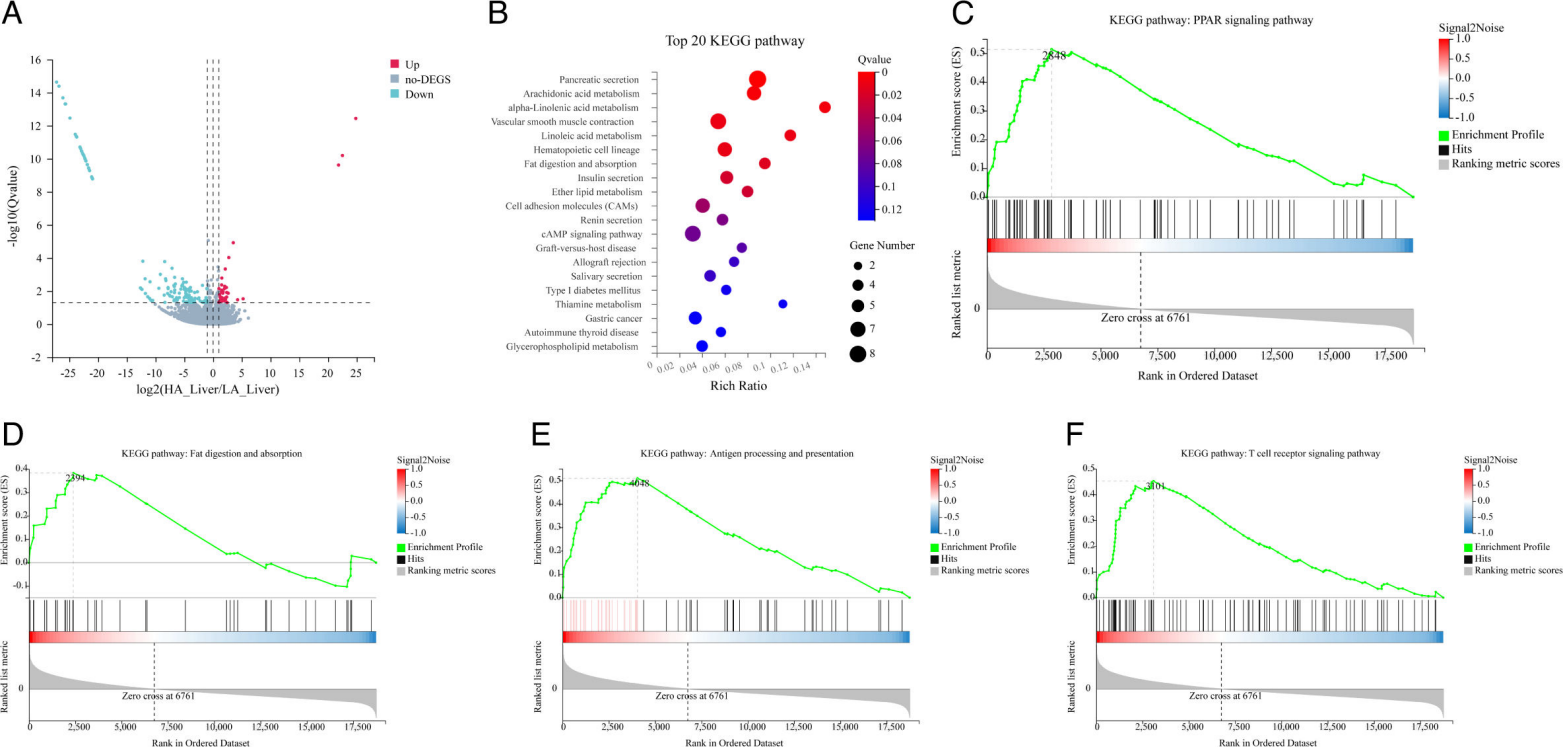
Transcriptome analysis of the liver in the low (LA) and high (HA) ADG groups. (**A**) Volcano plots indicating significant differentially expressed genes (DEGs) in the liver. (**B**) The top 20 enriched pathways of differential expressed genes (DEGs) between the LA and HA groups in the liver; transcriptome gene set enrichment analysis (GSEA) of the liver in the LA and HA groups; (**C**) enrichment plot of the PPAR signaling pathway gene set; (**D**) enrichment plots of the fat digestion and absorption gene set; (**E**) enrichment plots of the antigen processing and presentation gene set; (**F**) enrichment plots of the T cell receptor signaling pathway gene set. The criterion for the significantly affected KEGG pathway was NES >1, *P* value < 0.05, and FDR  <  0.25. NES, normalized enrichment score.

Based on a significance threshold of false discovery rate (FDR)   <   0.05 and Log2 (FC) ≥1, 91 annotated genes (33 upregulated and 58 downregulated) were found to be associated with ADG in the liver (Fig. 5A). A detailed list of all differentially expressed genes is shown in Table S6. To understand the biological functions of those DEGs, we performed a functional enrichment analysis. The results showed that those DEGs of the liver were mainly enriched in lipid metabolism, such as regulation of arachidonic acid metabolism, linoleic acid metabolism, and fat digestion and absorption (Fig. 5B). We also found the KEGG pathways involved in the immune system, including hematopoietic cell lineage, natural killer cell-mediated cytotoxicity, antigen processing and presentation, primary immunodeficiency, cytokine–cytokine receptor interaction, and T-cell receptor signaling pathway. Based on the results of the enrichment analysis, the differences in ADG between the LA and HA groups were inferred to be due to the metabolic efficiency and immune system. Subsequently, GSEA was performed on the two groups to avoid the deviation caused by single-gene analysis. Notably, GSEA indicated that the increased expression of the KEGG pathway in the HA group was primarily related to the energy metabolism process and immune system. These results provide further evidence that links alterations of these pathways to the difference in ADG. A list of sub-categories enclosed within each function is presented in Table S7. For the energy metabolism process, KEGG-base gene set enrichment analysis was enriched primarily for lipid and carbohydrate metabolism, including the PPAR signaling pathway (NES = 2.3563, *P* < 0.001, FDR < 0.001, Fig. 5C), fatty acid degradation (NES = 1.624, *P* = 0.018, FDR = 0.033, Fig. 5D), cholesterol metabolism (NES = 2.002, *P* < 0.001, FDR = 0.002), steroid biosynthesis (NES = 1.822, *P* < 0.001, FDR = 0.009), metabolism of xenobiotics by cytochrome P450 (NES = 1.673, *P* = 0.002, FDR = 0.022), regulation of lipolysis in adipocytes (NES = 1.7318, *P* = 0.005, FDR = 0.015), and glyoxylate and dicarboxylate metabolism (NES = 1.552, *P* = 0.026, FDR = 0.047). In addition, it was also closely related to the immune system, such as antigen processing and presentation (NES = 1.582, *P* < 0.001, FDR < 0.001, Fig. 5E), T cell receptor signaling pathway (NES = 1.582, *P* < 0.001, FDR < 0.001, Fig. 5F), natural killer cell-mediated cytotoxicity (NES = 2.586, *P* < 0.001, FDR < 0.001), hematopoietic cell lineage (NES = 1.582, *P* < 0.001, FDR < 0.001), and phagosome (NES = 1.582, *P* < 0.001, FDR < 0.001).

**Fig 6:**
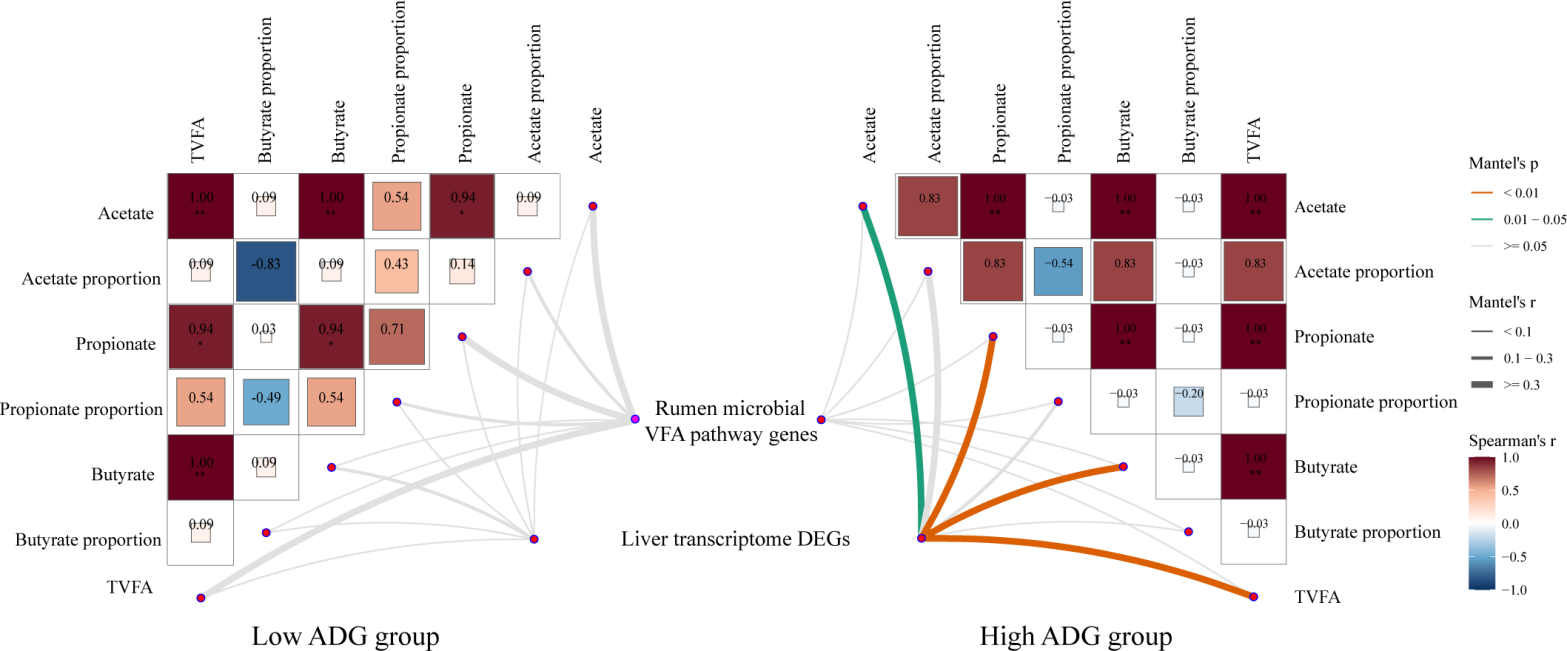
Links between rumen microbial genes, liver transcriptome, and host rumen VFA phenotypes in low ADG and high ADG lambs. The heatmap displays the relationships among rumen VFA profiles based on Spearman’s correlation analysis. The line indicates the relationship of the rumen microbial VFA pathway gene matrix and liver transcriptome matrix with the rumen VFA profile matrix based on the Mantel test. The line color indicates the *P* value in the Mantel test, and the thickness of the line indicates the correlation coefficient.

To further investigate the role of the VFA profile in ADG, we performed network association analyses among rumen microbial VFA functional genes, liver transcriptome DEGs, and rumen VFA profile. We observed that the liver transcriptome DEGs were strongly correlated with the levels of VFA in the high ADG group, including acetate, propionate, butyrate, and total VFA (Fig. 6).

## DISCUSSION

ADG is a key component in evaluating feed efficiency, which accounts for over 60% of the total phenotypic variation in the feed intake (12). A greater preweaning ADG would result in changes in epigenetic programming and enhance the growth performance (5). Therefore, considering ADG in weaning programs can help maximize efficiency. Additionally, understanding the underlying mechanisms responsible for high ADG lambs before weaning would be of great interest to express their utmost potential, enabling us to achieve more sustainable production. In the present study, lambs in the HA group had significantly higher ADG than those in the LA group. For systemic nutrient metabolism, the AA profile is a key indicator of protein turnover in the host, which represents the sum of the dynamic metabolic flow of nutrients and metabolites from all tissues and organs (13). The increase in several serum-free AAs in the HA group of this study is consistent with the vital role of amino acids in maximizing the growth rate and muscle growth (14).

Given that VFAs derived from the rumen can supply up to 70% of the net energy requirements of the animal (15), evaluating the VFA metabolism between the low and high ADG groups allows us to obtain a better insight into their phenotype divergence. The accumulated evidence indicated that differences in the VFA concentration between low and high ADG groups were inconsistent among studies (16–18). These inconsistencies can be attributed to variations in diet, breed, age, and feed intake, as well as sample collection. In the present study, no differences were found in total VFA, acetate, propionate, and butyrate concentration between the two groups. However, the HA group had a higher molar proportion of propionate and a lower ratio of acetate to propionate, which is in line with the finding in a recent study in Hu sheep (19). As one of the fermentation products in the rumen, propionate is well-recognized as a primary precursor of gluconeogenesis in ruminants, which could be transported across the rumen epithelium, converted into glucose in the liver, and accounted for 54% of the glucose used by the animal (20). These findings indicate that lambs in the high ADG group shifted their rumen fermentation pattern toward glucogenic propionate production (lower A/P ratio), resulting in improved feed energy utilization efficiency for growth and, subsequently, higher ADG.

During the weaning age, the rumen ecosystem is key in attaining higher growth rates and better health later in life (15, 21, 22). Hence, the rumen microbiota is a potential target for improving ruminant production and animal health. However, there were no major shifts between the LA and HA groups in the current study when evaluating OTU alpha and beta diversity and the relative abundance of bacteria taxa at the genus level or higher taxonomic ranks, suggesting no prime shift between the two ADG groups in the ruminal bacterial communities and population structures. The dominance of Firmicutes and Bacteroidetes in the rumen of ruminants has been widely reported in previous studies (23, 24). In line with those studies, Firmicutes and Bacteroidetes were identified as the most dominant phyla in our study. Specifically, many of the changes were identified within the phylum Firmicutes between different efficient animals, which has been shown to affect energy harvest (25). The relative abundance of Firmicutes in the HA group was greater than that in the LA group (LA vs HA: 34.59% vs 41.59%), which may be attributed to energy efficiency. Previous studies have commonly reported that several rumen bacteria, such as *Prevotella* and *Ruminococcus*, were associated with feed efficiency traits, including ADG and feed intake (26, 27), and the lack of significant changes in 16S rRNA between the ADG phenotypes was unexpected. This discrepancy between studies can be explained by several key aspects. In the present study, the lambs were breastfeeding and followed the same progressive weaning process with the same starter at a similar level of intake. Therefore, some factors, such as diet and feed intake, which might affect the rumen microbial population, were avoided. It would explain the paucity of differences between the two groups in ruminal microbiota.

During the preweaning period, the lamb harbors a highly variable and increasingly complex microbial community, which is easily disrupted (28). The genes annotated by metagenomic sequencing were compared with KEGG databases to obtain the annotation information of metabolic pathways, and the influence of different growth rates on rumen microflora function was analyzed, especially the propionate production pathway. Three different biochemical pathways for propionate production are known to be present in the microbiota: succinate pathway, acrylate pathway, and propanediol pathway (29, 30). Meanwhile, propionate is commonly converted from the succinate pathway in the intestine (31, 32). In the present study, the abundance of enzyme genes sdhA and PCT was greater in the high ADG group, which might be the main reason for greater propionate production in the high ADG group. The enzyme gene sdhA encodes the succinate dehydrogenase complex, which converts fumarate into succinate. It could account for the enhancement of propionate synthesis. Although the abundance of PCT genes encoding the propionyl-CoA-transferase was low, it is the rate-limiting enzyme that is responsible for the last step in the propionate synthesis (33). Hence, as stated previously, the increased rumen propionate is more likely due to changes in rumen microbial function; thus, the increase in abundances of enzyme genes sdhA and PCT can be attributed to the high efficiency of lambs from the HA group via the succinate pathway.

The liver serves as the ultimate arbiter of nutrients available to the periphery in support of growth and must adapt to patterns of absorbed nutrients. Increasing evidence suggests that energy and lipid metabolism in the liver are the key factors affecting feed utilization, which might be the potential mechanism of different growth rates in livestock and poultry (34–36). In support of those findings, pathways and processes related to energy metabolism were overrepresented in lambs from the HA group. The PPAR signaling pathway and fatty acid degradation were upregulated, which provides more compensatory precursors (acetyl-CoA) for ATP synthesis (37). PPARs are known to be involved in the uptake, oxidation, and storage of fatty acids (38). The rate of the TCA cycle depends on the concentrations of oxaloacetate and acetyl-CoA in the first step (39, 40). With the close collaboration of the PPAR signaling pathway, fatty acids are transported into the mitochondria, undergo oxidative phosphorylation, and generate acetyl-CoA via beta-oxidation. Meanwhile, the increased ruminal propionate is transported to the liver to promote gluconeogenesis and oxidation of acetyl CoA (41). Subsequently, the increased usability of acetyl-CoA facilitates citrate oxidation, which is a crucial energy fuel in the TCA cycle (42). On the other hand, glyoxylate metabolism and dicarboxylate metabolism, as the primary metabolism of carbon metabolism, were overrepresented in HA lambs. As a vital assistant in the TCA cycle, glyoxylate metabolism and dicarboxylate metabolism were elevated in response to the high growth rate, suggesting that the carbon metabolism was promoted and the TCA cycle was improved indirectly (43, 44). In summary, activated fatty acid degradation and glyoxylate and dicarboxylate metabolism in the high ADG group provide more precursors and metabolic intermediates in the TCA cycle to promote ATP synthesis than those in the LA group. In addition, the network association analysis indicated that the VFA profile plays a key role in the energy supply linking the liver transcriptome and increased ADG in the HA group before the weaning period.

Due to the lifetime effect on the growth trajectory, animals with poor preweaning ADG have lower growth potential; thus, the transition phase of the lambs is the critical window period for manipulating visceral organ development and metabolic processes. The process of rumen development is a major challenge before weaning relating to the morphological development of rumen papillae, functional achievement in fermentation and metabolism, and microbial colonization (45). Given that no DEGs in the rumen transcriptome were noticed between the two groups, we used GSEA to evaluate the upregulated KEGG pathways involved in rumen epithelial growth and immune response in the HA group. It is well known that the JAK-STAT pathway is a principal signaling mechanism for a multitude of cytokines and growth factors, which stimulate immune function, cell proliferation, differentiation, migration, and apoptosis (46). The pleiotropic cross-talk between the JAK-STAT pathway and other pathways (PI3K/AKT/mTOR signaling, Wnt signaling, and NF-kappa B signaling) has an important role in the development and homeostasis of the host (47). The interaction between metabolite–gut–organ axes will also affect the immune system and host physiology. In the present study, enhanced rumen epithelium immune function could be due to immune cell development and differentiation caused by the Th17 cell differentiation and T cell receptor signaling pathway. Responding to the high ADG, the immune pathway in the liver was also activated for disease resistance and immune defense, including natural killer cell-mediated cytotoxicity, antigen processing and presentation, hematopoietic cell lineage, phagosome, and T cell receptor signaling pathway, which could have a further impact on animal health status (48). Paradis et al. reported that heifers with a stronger hepatic innate immunity or a larger population of immune cells could lead to a rapid response to stimulus, and, therefore, spend less energy to fight inflammation and leave more energy for growth (49). Concurrently, the liver transcriptome DEGs were strongly correlated with VFA concentration, indicating that the VFA profile plays a key role in the energy supply linking the liver transcriptome and increased ADG before the weaning period. In the combined transcriptome analysis of two tissues and correlation analysis, the rumen paid more attention to its own growth and development before weaning, while the liver played a major role in driving the shift in ADG through the energy metabolism.

### Conclusion

**Fig 7:**
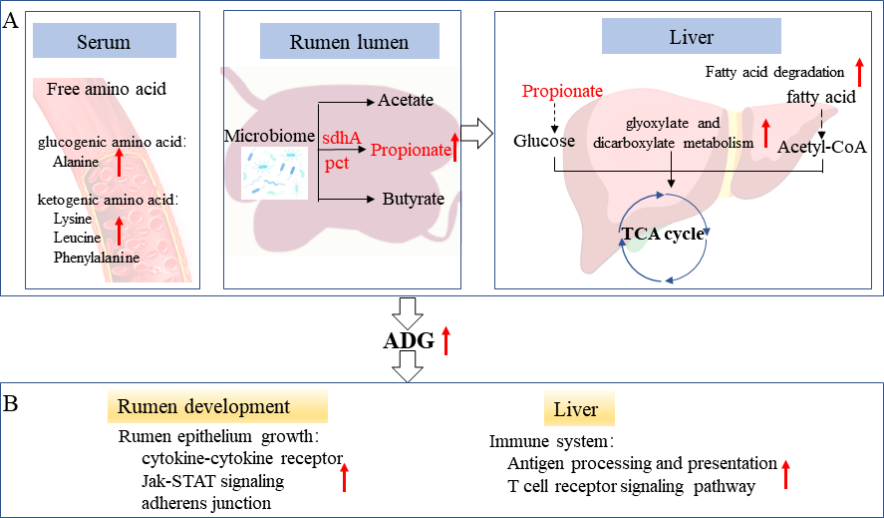
Integrative diagram showing the effects and potential mechanisms in the preweaning lambs with divergent ADG. (**A**) Comprehensive response of rumen microbiota and liver transcriptome to different ADG in preweaning lambs. Lambs from the HA group promoted serum AA production, enhanced the expression of enzyme genes sdhA and PCT in the propionate production pathway, and activated energy production via the TCA cycle in the liver to support host growth in high ADG. (**B**) Lambs with high ADG might benefit for rumen development and liver metabolic and immune function and thus optimized growth potential. The red font represents upregulated microbiota, genes, and biological processes in high ADG lambs.

No significant difference was observed in body weight change in preweaning lambs among the four groups. However, the ADG variation analysis for individual lambs provided an insight into the regulated mechanisms of increased growth rate before weaning and its potential long-term effects on the host (Fig. 7). The lambs with high ADG had greater abundances of genes sdhA and PCT in the propionate production pathway, illustrating that the increased propionate production and decreased acetate: propionate ratio was definitely related to the succinate pathway. Meanwhile, enhanced activated fatty acid degradation and glyoxylate and dicarboxylate metabolism in the liver provided more fuel into the TCA cycle, which might be the main mechanism of high efficiency. These results indicated that efficient lambs enhanced microbial VFA metabolism toward rumen propionate and serum AA production to support host growth. Furthermore, high ADG benefits for the development of the rumen epithelium in the preweaning period, which could have a profound influence on nutrient digestion and absorption. The enriched immune function in the high ADG group suggested that the activated immune pathway in the liver may help with disease resistance and host defense, leading to better growth later in life.

## Data Availability

Raw reads of 16S rRNA gene sequencing of ruminal microbiota are available at the National Center for Biotechnology Information (NCBI) Sequence Read Archive (SRA) (project number PRJNA972526). Raw reads of metagenomic sequencing of rumen content are available at NCBI SRA (project number PRJNA972992). Raw reads of transcriptome sequencing of the ruminal epithelium and liver are available at NCBI SRA (project number PRJNA972631).

## SUPPLEMENTAL MATERIAL

Figure S1 msystems.00873-24-s0001.tif Experiment design.

Figure S2 msystems.00873-24-s0002.tif Microbial KEGG pathway.

Legends msystems.00873-24-s0003.docx Legends for Fig. S1 and S2.

Supplemental tables msystems.00873-24-s0004.xlsx Tables S1 to S7.

ASM does not own the copyrights to Supplemental Material that may be linked to, or accessed through, an article. The authors have granted ASM a non-exclusive, world-wide license to publish the Supplemental Material files. Please contact the corresponding author directly for reuse.
