# Supplementary figures and images for: Ruminal microbial metagenomes and host transcriptomes shed light on individual variability in the growth rate of lambs before weaning: the regulated mechanism and potential long-term effect on the host
Source: mSystems. 2024 Aug 20;9(9):e00873-24. doi: 10.1128/msystems.00873-24 (PMC11406974; doi:10.1128/msystems.00873-24)

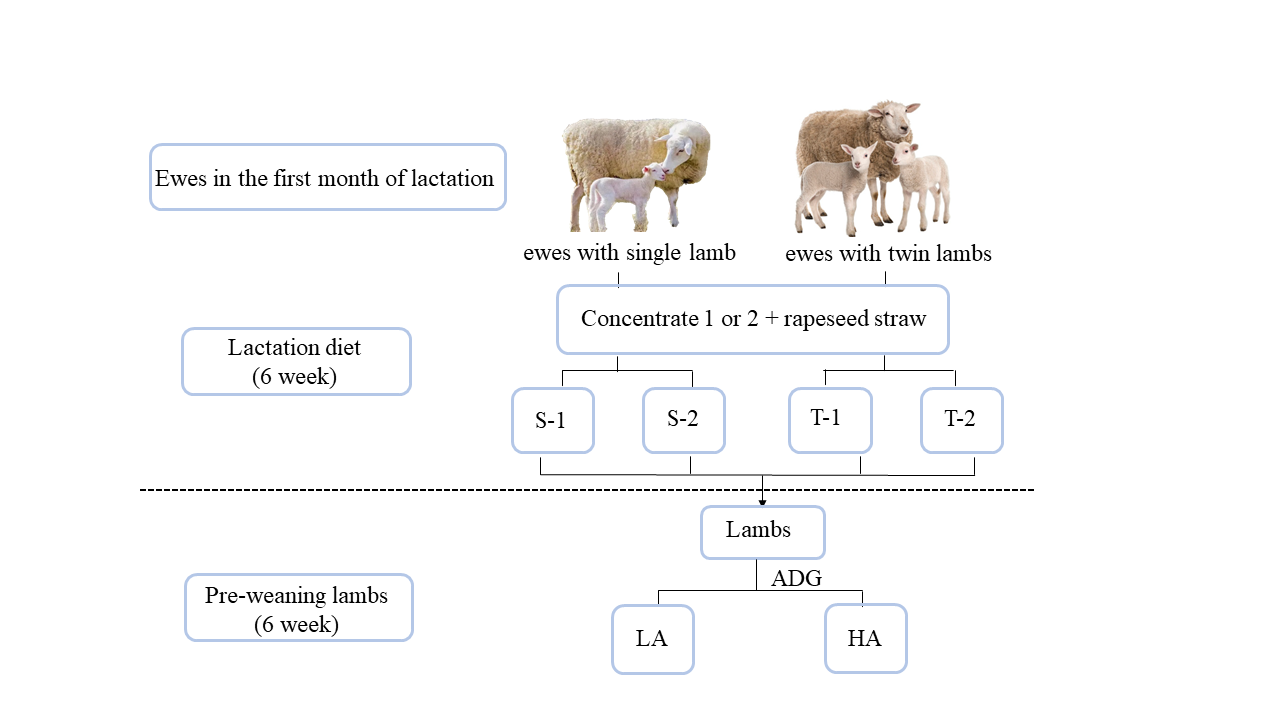

Supplement: Figure S1 — Experiment design. [file msystems.00873-24-s0001.tif]

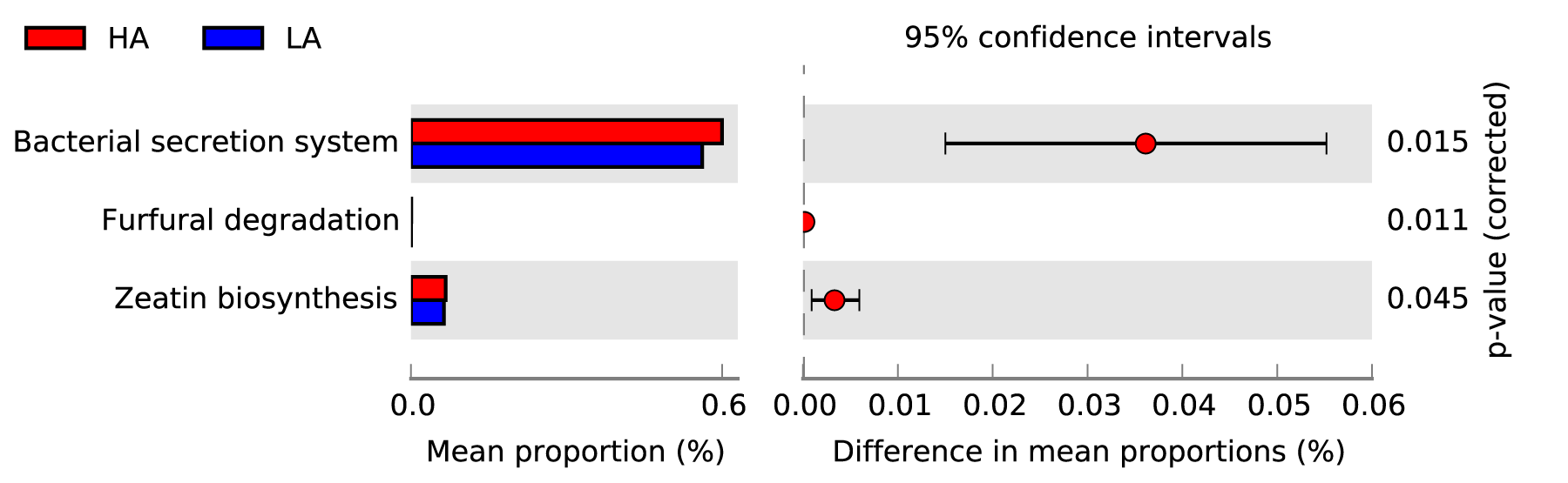

Supplement: Figure S2 — Microbial KEGG pathway. [file msystems.00873-24-s0002.tif]
