# Supplementary material for: Ruminal microbial metagenomes and host transcriptomes shed light on individual variability in the growth rate of lambs before weaning: the regulated mechanism and potential long-term effect on the host
Source: mSystems. 2024 Aug 20;9(9):e00873-24. doi: 10.1128/msystems.00873-24 (PMC11406974; doi:10.1128/msystems.00873-24)
Supplement: Legends — for Fig. S1 and S2. [file msystems.00873-24-s0003.docx]

**Fig S1** The flow chart of the experimental design and treatment groups. Twenty Hulunbeir lactating ewes rearing single or twin lambs were raised with two different concentrates and allocated into 4 groups: T-1 = lactating ewes rearing twin lambs fed with concentrate diet 1 and rapeseed straw, n=5; S-1 = lactating ewes rearing single lamb fed with concentrate diet 1 and rapeseed straw, n=4; T-2 = lactating ewes rearing twin lambs fed with concentrate diet 2 and rapeseed straw, n=7; S-2 = lactating ewes rearing single lamb fed with concentrate diet 2 and rapeseed straw, n=4. The experiment began when the lambs were one-month and lasted for 6 weeks. All the experimental lambs were weighed before morning feeding at the beginning and last day of the experimental period, and the average daily weight gain (ADG) was calculated. According to the rank of ADG, 12 most extreme preweaning lambs were allotted into high (HA, n = 6) and low (LA, n = 6) ADG groups.

**Fig S2** The Significantly different KEGG pathways level3 in metagenome analysis between the low (LA) and high (HA) ADG groups.
